# Comparative Analysis of Fecal Microbiota Between Adolescents with Early-Onset Psychosis and Adults with Schizophrenia

**DOI:** 10.3390/microorganisms12102071

**Published:** 2024-10-16

**Authors:** Lucero Nuncio-Mora, Humberto Nicolini, Nuria Lanzagorta, Cynthia García-Jaimes, Fernanda Sosa-Hernández, Vanessa González-Covarrubias, Héctor Cabello-Rangel, Emmanuel Sarmiento, David C. Glahn, Alma Genis-Mendoza

**Affiliations:** 1 Posgrado en Ciencias Biológicas, Unidad de Posgrado, Edificio D, 1° Piso, Circuito de Posgrados, Ciudad Universitaria, Coyoacán, Ciudad de México 04510, Mexico; lu.nunciom@gmail.com; 2 Laboratorio de Genómica de las Enfermedades Psiquiátricas y Neurodegenerativas, Instituto Nacional de Medicina Genómica, Secretaría de Salud, Ciudad de México 14610, Mexico; hnicolini@inmegen.gob.mx; 3 Grupo Médico Carracci, Departamento de Investigación Clínica, Ciudad de México 03740, Mexico; lanzagorta_nuria@gmc.org.mx (N.L.); cynth8a@gmail.com (C.G.-J.); fer.sosaher98@gmail.com (F.S.-H.); 4 Laboratorio de Farmacogenómica, Instituto Nacional de Medicina Genómica, Secretaría de Salud, Ciudad de México 14610, Mexico; vgonzalez@inmegen.gob.mx; 5 Hospital Psiquiátrico Fray Bernardino Álvarez, Servicios de Atención Psiquiátrica, Secretaria de Salud, Ciudad de México 14080, Mexico; hector19.05.19.05@gmail.com; 6 Instituto Nacional de Psiquiatría Juan Ramón de la Fuente Muñiz, Secretaría de Saludos, Ciudad de México 14370, Mexico; emmanuelsarmientoh@hotmail.com; 7 Department of Psychiatry and Behavioral Sciences, Boston Children’s Hospital, Boston, MA 02115, USA; david.glahn@childrens.harvard.edu; 8 Department of Psychiatry, Harvard Medical School, Boston, MA 02215, USA; 9 Olin Neuropsychiatry Research Center, Institute of Living, Hartford, CT 06106, USA; 10 Hospital Psiquiátrico Infantil Dr. Juan N. Navarro, Servicios de Atención Psiquiátrica, Secretaria de Salud, Ciudad de México 14080, Mexico

**Keywords:** gut microbiota, early onset psychosis, schizophrenia, 16S rRNA sequencing, adolescents, gut–brain axis

## Abstract

Studies of the composition of the gut microbiome have consistently shown that psychiatric disorders such as schizophrenia are associated with gut dysbiosis. However, research focusing on adolescents with early-onset psychosis remains limited. This study aimed to characterize the microbial communities and their potential metabolic functions in these populations. We identified that genera *Desulfovibrionaceae_Incertae_Sedis*, *Paraprevotella*, and several genera from the Oscillospiraceae family were significantly more abundant in patients with schizophrenia compared to non-psychotic individuals, while *Dorea* showed decreased levels in schizophrenia patients. Furthermore, patients with early-onset psychosis demonstrated a significant reduction in *Staphylococcus* abundance. Additionally, we observed an increase in *Prevotellaceae Leyella* and *Prevotellaceae Incertae Sedis* in patients receiving atypical antipsychotic treatment, along with a rise in the genus *Weissella* among those treated with sertraline. Conversely, patients on valproate treatment exhibited decreased levels of *Desulfovibrionaceae Incertae Sedis*, while showing increased levels of *Kandleria* and *Howardella*. Functional prediction analysis using PICRUSt2 revealed significant differences in the expression of key enzymes associated with fatty acid metabolism. Gene orthology analysis identified 10 differentially expressed genes in the early-onset psychosis and schizophrenia groups. Our findings underscore the importance of considering dietary factors, pharmacological treatments, and microbial composition in understanding the gut–brain axis in psychiatric disorders.

## 1. Introduction

Psychosis is a neuropsychiatric disorder associated with delusions, hallucinations, disorganized thinking and confusion. The psychotic episode should last no longer than three months, and the symptoms described above should not be the result of any drug or medical condition [1,2].

The average age at which the first psychotic episode occurs is usually around 20 years of age, but early-onset psychosis can occur before the age of 18, which is usually associated with functional impairment [1,3]. Schizophrenia is the most common psychiatric disorder, with prevalence worldwide between 0.5% and 1.5% [4]. It is associated with persistent psychotic symptoms. However, psychosis can also manifest in other conditions such as major depressive disorder, bipolar disorder, schizoaffective disorder, delusional disorder, and schizophreniform disorder [5]. Intensive research on psychiatric disorders has pinpointed genetic, clinical, familial, and other factors that are strongly correlated with the disease. For instance, obstetric complications, maternal viral infections, malnutrition, drug and stress exposure, childhood trauma, and heritability may all contribute to an increased risk of developing schizophrenia [6]. More recently, the gut microbiome has been shown to play a critical role in the development of several diseases. It has been suggested that the gut–brain axis impacts the development of psychiatric disorders such as bipolar disorder, depression, anxiety, eating disorders and schizophrenia [7].

The gut microbiota is composed of a diversity of microorganisms, including bacteria, viruses, fungi, and archaea, which interact with each other and with the host through several mechanisms [8]. It is estimated that the intestine contains between 1000 and 5000 species, the majority of which belong to the phyla Firmicutes, Bacteroidetes, Actinobacteria, Proteobacteria, Fusobacteria, and Verrucomicrobiota [9].

The role of the gut microbiome is diverse; it can metabolize our food, synthesize essential vitamins and nutrients, prime the immune system, and protect us from infections. It also alters the pharmacokinetics and pharmacodynamics of hundreds of drugs, either affecting their metabolism, elimination, or altering the levels of drug targets [10,11]. It has been acknowledged that the bidirectional communication between the intestine and the brain is facilitated through different pathways such as the vagus nerve, the endocrine system, and the immune system [12].

Despite advances in research, the pathogenesis of both schizophrenia and early-onset psychosis remains incomplete, and current studies cannot explain its high interindividual variability. In addition, clinically, there are no validated biomarkers that could aid in the diagnosis of schizophrenia and early-onset psychosis; current practices can be somehow subjective through the identification of the patients’ symptoms [13,14].

It is evident that more efficient biomarkers and therapies are urgently needed and that additional clinical factors ought to be considered since the current ones cannot fully diagnose nor support a comprehensive and definitive treatment, hence the interest in investigating the gut microbiome. Currently, there are only a few studies on the early onset of psychosis in adolescents that consider the microbiome or its potential impact for early diagnosis; it is relevant to define the microbiome’s identity and functionality to better explain the evolution of early-onset psychosis. Therefore, the aim of this study was to determine the composition of the gut microbiota in adolescents with early-onset psychosis and compare it with non-psychotic patients and patients with schizophrenia.

## 2. Materials and Methods

### 2.1. Study Participants

Twenty-one non-psychotic patients, 12 patients with early-onset psychosis, and 15 patients with schizophrenia were recruited between December 2021 and July 2023 at the Hospital Psiquiátrico Infantil “Dr. Juan N. Navarro” in Mexico City. Patients were evaluated and diagnosed by a specialized psychiatrist recruited using the MINI-KID test. Inclusion criteria for the early-onset psychosis group were (1) patients aged 10 to 18 years, (2) meeting the criteria for psychosis as determined by the MINI-KID, (3) hallucinations or delusions were not due to substance use or other illness, (4) no use of antibiotics for at least 3 months prior to stool sampling, and (5) no surgical procedures such as gastroscopy and colonoscopy (within the last 3 months) or any major gastrointestinal surgery for at least 5 years. For non-psychotic patients, the inclusion criteria were the same except that they did not meet lifetime criteria for psychosis, although they could be receiving treatment for another psychiatric disorder such as depression, anxiety, and attention-deficit/hyperactivity disorder (ADHD).

Patients with schizophrenia were evaluated using the positive and negative syndrome scale (PANSS) at the Fray Bernardino Psychiatric Hospital. Inclusion criteria were (1) patients aged between 20 and 70 years, (2) no use of antibiotics for at least 3 months prior to stool sampling, and (3) no surgical procedures such as gastroscopy and colonoscopy (in the last 3 months) or major gastrointestinal surgery in at least 5 years.

All participants signed an informed consent and assent form, as appropriate. The research protocol was approved by the Research Ethics Committee of the Hospital Psiquiátrico Infantil “Dr. Juan N. Navarro” (CONBIOETICA 09-CEI-013-20160708) and the Hospital Fray Bernardino Álvarez in accordance with the Declaration of Helsinki.

### 2.2. Fecal Sample Collection

Stool samples were collected in a stool collection bottle at the participant’s home following an instruction manual with an in-house kit, kept in the fridge, and delivered to the investigator between 24 and 48 h. Samples were aliquoted and stored at −80 °C until use.

### 2.3. DNA Extraction

DNA extraction from feces was performed using the commercial kit QIAmp PowerFecal pro (Qiagen, Hilden, Germany) according to the manufacturer’s specifications. DNA was evaluated for purity and concentration in a NanoDrop 2000 c spectrophotometer (ThermoFisher Scientific Waltham, MA, USA). For each sample, the absorbance index (A260/280) was measured as an indicator of protein contamination, and only samples between 1.8 and 2.0 were considered. DNA integrity was determined by 1% agarose gel electrophoresis. Finally, the DNA was stored at −20 °C until sequencing.

### 2.4. Amplification

The bacterial region V3-V4 of the 16S rRNA gene was amplified using the 16S V3 (341F) forward and V4 (805R) reverse primers and adapters from Illumina following the manufacturer’s 16S metagenomic sequencing library protocol. Library quality control was verified by microcapillary electrophoresis in a TapeStation 4200 (Agilent, Technologies, Santa Clara, CA, USA); libraries were normalized and pooled to 10.2 nM, denatured, and diluted to 10 pM. Sequencing was performed using a 2 × 250 bp cartridge/MiSeq Reagent Kit V3 in an MiSeq Illumina sequencer (Illumina, San Diego, CA, USA). Next-generation sequencing was performed at INMEGEN at the Sequencing Unit in a MiSeq NGS sequencer (Illumina, San Diego, CA, USA).

### 2.5. Statistical Analysis of Clinical Characteristics

To compare clinical characteristics between groups, we performed Kruskal–Wallis tests for continuous variables such as age, BMI, and years of education. Pos-hoc pairwise comparisons were conducted using Dunn’s test with Benjamini–Hochberg (BH) correction for multiple comparisons. Sex ratio differences between groups were analyzed using a Chi-square test.

### 2.6. Bioinformatic Analyses

Raw FASTQ files (R1 and R2) were processed and analyzed using the QIIME2 (Quantitative Insights Into Microbial Ecology 2) 2024.5 program [15]. The 250 bp reads were truncated at any site of quality score of 20. Dada2 (version 2024.5.0) was used to join the R1 and R2 sequences and to filter and remove chimeras from them [16]. All amplicon sequence variants (ASVs) were aligned using the mafft program (version 2024.5.0) [17] and used to construct a phylogeny.

After sample rarefaction (26500 sequences per sample), alpha diversity analysis was performed using the phyloseq package (version 1.44.0) [18] and vegan package (version 2.6-8) [19] in R 4.3.1 [20]. Group differences were assessed using the Wilcoxon test, with Benjamini–Hochberg (BH) correction applied.

Beta diversity was estimated using Bray–Curtis, Jaccard, unweighted UniFrac, and weighted UniFrac distance matrices. Pairwise permutational multivariate analysis of variance (pairwise PERMANOVA) with BH correction was conducted to assess group variance using the adonis function from the vegan package (version 2.6-8) [19], with non-psychosis, early-onset psychosis, and schizophrenia as the explanatory variables. Principal coordinates analysis (PCoA) was performed for each distance matrix.

Taxonomy assignment of amplicon sequence variants (ASVs) was conducted using the SILVA 138.2 database pre-trained with the classify—Sklearn Naïve Bayes classifier (version 2024.5.0) [21,22] as a reference. Differences in the relative abundance of taxa were evaluated using the MaAsLin2 package (version 1.14.1) [23], with the following formula to control for covariates: DX + Age_Group + BMI + Sex+ Atypical_antipsychotics + Sertraline + Methylphenidate + valproate + Benzodiazepines + DX_Age_Group.

The q2-picrust2 plugin was used to infer enzyme commission (EC) numbers, KEGG orthology (KOs), and MetaCyc pathways based on the 16S rRNA V3-V4 region from our study [24]. Subsequently, ANCOMBC (version 2.2.2) [25,26] was applied to compare functional enrichment in these pathways and correlate them with the diagnoses of non-psychosis, early-onset psychosis, and schizophrenia patients.

## 3. Results

### 3.1. Clinical Data

Statistically significant differences in age and education level were found between patients with schizophrenia and non-psychotic patients and patients with early-onset psychosis. There were no significant differences in gender and BMI (Table 1).

### 3.2. Sequencing Data

A total of 7,622,765 raw sequences were identified for both forward and reverse reads in all 48 samples processed. After filtering and chimera removal, 2,309,798 amplicon sequence variants (ASVs) were obtained; the average number of ASVs per sample was 50,935 reads (min 26,561; max 62,328) and considered for further bioinformatic analyses in the Software QIIME2 (version 2024.5) [15].

### 3.3. Alpha Diversity

**Figure 1:**
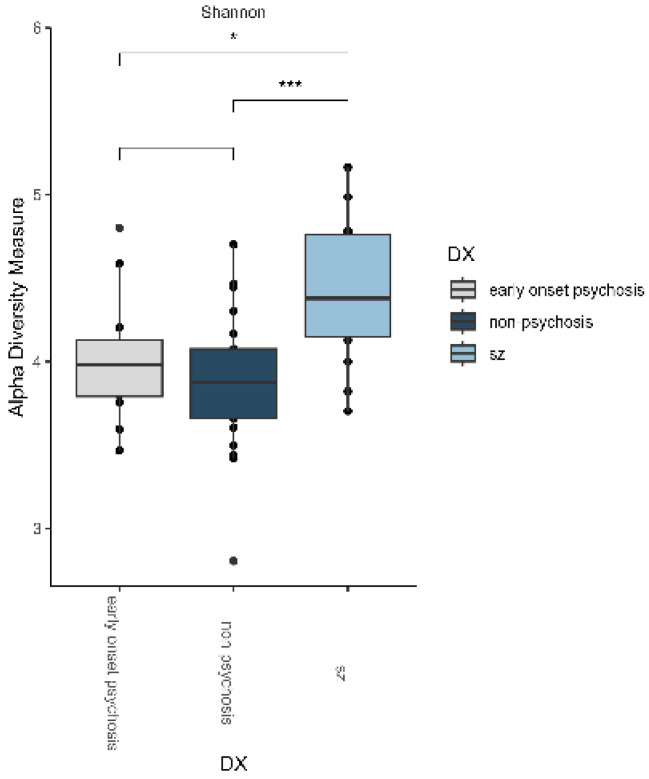
The alpha diversity analysis revealed that patients with schizophrenia (SZ) exhibited significantly higher microbial richness and diversity, as measured by the Shannon index (* *p* < 0.05, *** *p* < 0.01), compared to both the non-psychosis and early-onset psychosis groups. Asterisks denote groups that are statistically different, as determined by the Wilcoxon test with Benjamini–Hochberg (BH) correction.

Bacterial richness and diversity were characterized using multiple metrics. For alpha diversity, we assessed the observed, Chao1, Shannon, and Simpson indices (Supplementary Figure S1). Of these, only the Shannon index showed significant differences between patients with schizophrenia (SZ) and both non-psychosis and those with early-onset psychosis patients (Supplementary Table S1, Figure 1). In contrast, no statistically significant differences (*p* > 0.05) were found between non-psychosis and those with early-onset psychosis patients (Supplementary Table S1, Figure 1).

### 3.4. Beta Diversity

**Figure 2:**
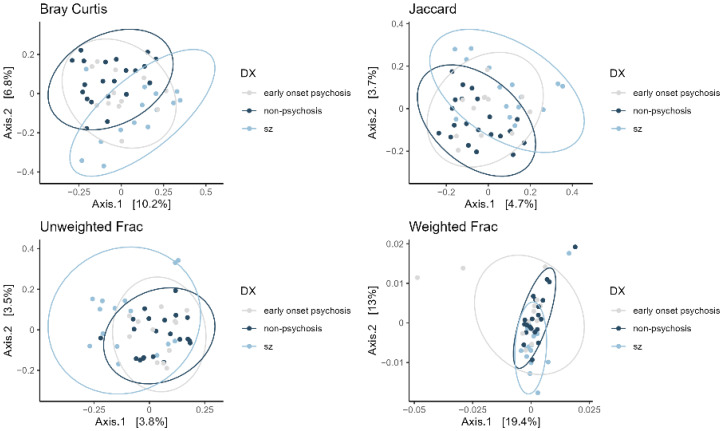
Beta diversity indices, Bray Curtis, Jaccard, Weighted Frac, and Unweighted Frac assessing the homogeneity of the bacterial communities of the non-psychosis, early-onset psychosis, and schizophrenia groups.

The beta diversity analysis compares bacterial abundance and richness between study groups, describing the dissimilarity of bacterial communities across different groups. This was assessed using the metrics: Bray Curtis, Jaccard, Weighted Frac, and Unweighted Frac indexes. In all four indexes, we observed greater homogeneity between the non-psychotic and early-onset psychotic groups. In contrast, the bacterial community in patients with schizophrenia showed significantly different homogeneity compared to the other two groups (pairwise PERMANOVA, *p* < 0.05, Supplementary Tables S2 and S5, Figure 2). Particularly, for the weighted and unweighted UniFrac indexes, we observed significantly greater heterogeneity in the bacterial community between the schizophrenia and non-psychotic groups (Supplementary Tables S4 and S5).

### 3.5. Composition of the Gut Microbiome and Differences Between Study Groups

**Figure 3:**
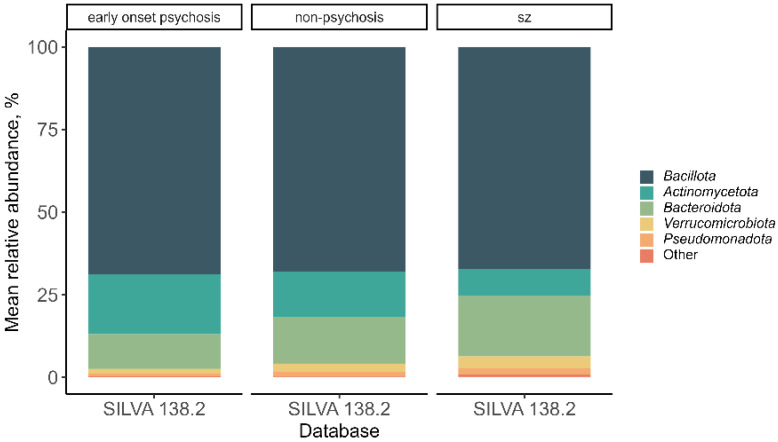
Mean relative abundance of phyla distributed by non-psychosis, early-onset psychosis, and schizophrenia. The group “Other” refers to the phyla with a mean relative abundance <5%.

All groups showed 16 different phyla. Among them, Bacillota was the most abundant phylum in all three groups, followed by Actinomycetota, Bacteroidota, Verrucomicobiota, and Pseudomonadota (Figure 3).

**Figure 4:**
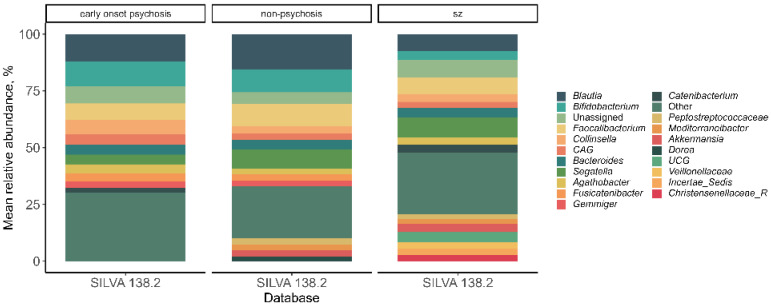
Mean relative genre abundance classified in early-onset psychosis, non-psychosis, and schizophrenia (sz) groups. The group “Other” refers to the genre with a mean relative abundance <2%.

**Figure 5:**
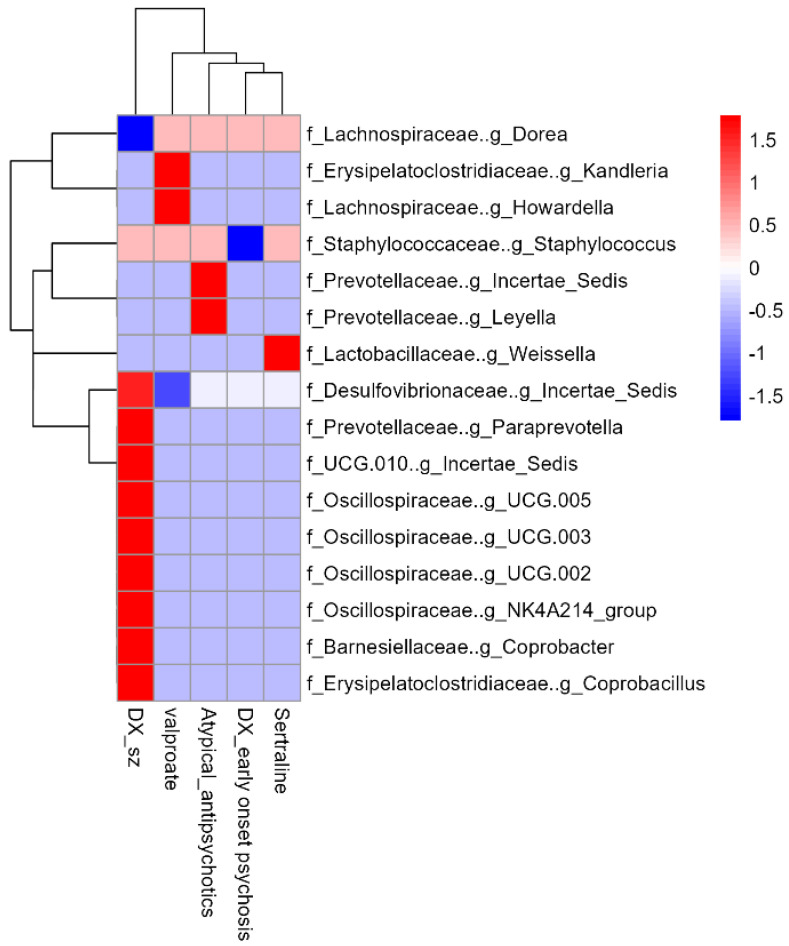
Heatmap of differential relative abundance of bacterial genera between non-psychotic patients and those with early-onset psychosis and SZ, adjusted for age, atypical antipsychotics, valproate, and sertraline.

At the genus level, a total of 236 different genera were identified, of which 21 exhibited a relative abundance greater than 2% (Figure 4). Differences in relative abundance among the three patient groups were analyzed, finding that *Desulfovibrionaceae_Incertae_Sedis*, *Paraprevotella*, *UCG_010*, *Oscillospiraceae_UCG-005*, *Oscillospiraceae_UCG-003*, *Oscillospiraceae_UCG-002*, *Oscillospiraceae_NK4A214*, *Coprobacter*, and *Coprobacillus* showed higher abundance in patients with schizophrenia compared to those without psychosis (MaAsLin2, *p* < 0.05). Additionally, a decrease in Dorea was observed in patients with schizophrenia compared to those without psychosis. In contrast, Staphylococcus showed a significant decrease in patients with early-onset psychosis compared to non-psychotic patients (MaAsLin2, *p* < 0.05) (Figure 5).

Additionally, a significant increase in *Prevotellaceae Leyella* and *Prevotellaceae Incertae Sedis* was observed in patients receiving treatment with atypical antipsychotics. Likewise, those undergoing treatment with sertraline showed an increase in the genus *Weissella*. On the other hand, patients treated with valproate exhibited a decrease in *Desulfovibrionaceae Incertae Sedis*, as well as an increase in *Kandleria* and *Howardella*.

### 3.6. Functional Characterization of Microbiota Between Study Groups

**Figure 6:**
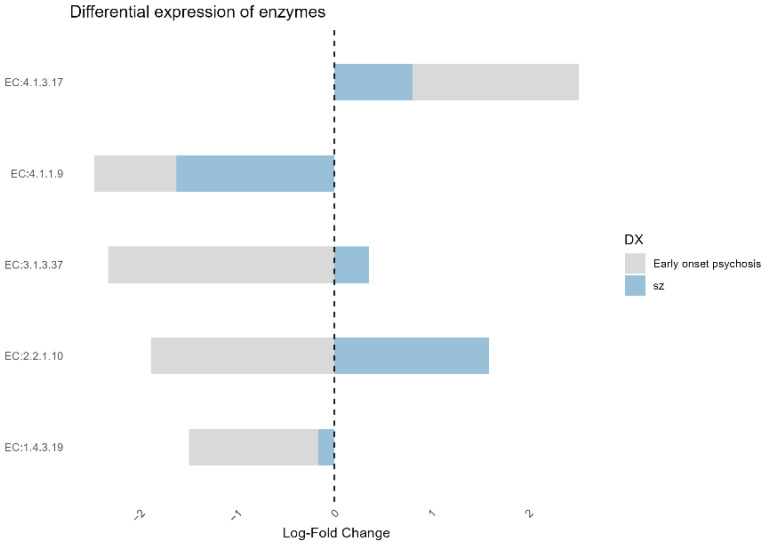
Barplot showing the differential expression (log-fold change) of EC numbers in patients with early-onset psychosis and schizophrenia compared to non-psychotic. The log-fold change indicates the relative abundance of specific enzymes (EC numbers) between the groups, highlighting key metabolic differences between the conditions.

To investigate the possible metabolic and biological functions of the microbial communities in our study groups, functional prediction analysis was performed using PICRUSt2. The differential analysis with ANCOMBC identified five enzyme commission (EC) numbers with significantly different expressions between the groups. The enzymes EC 4.1.3.17 (4-hydroxy-4-methyl-2-oxoglutarate aldolase), EC 3.1.3.37 (sedoheptulose-bisphosphatase), and EC 2.2.1.10 (2-amino-3,7-dideoxy-D-threo-hept-6-ulosonate synthase) showed higher abundance in patients with schizophrenia compared to the non-psychotic group. Conversely, EC 4.1.1.9 (malonyl-CoA decarboxylase), EC 3.1.3.37 (sedoheptulose-bisphosphatase), EC 2.2.1.10 (2-amino-3,7-dideoxy-D-threo-hept-6-ulosonate synthase), and EC 1.4.3.19 (glycine oxidase) exhibited a significant decrease in patients with early-onset psychosis compared to the non-psychotic group (Figure 6). It is important to highlight that both patients with early-onset psychosis and those with schizophrenia shared a differential pattern in enzymes EC 4.1.1.9 and EC 1.4.3.19, with lower abundance, and EC 4.1.3.17, with higher abundance (Figure 6).

**Figure 7:**
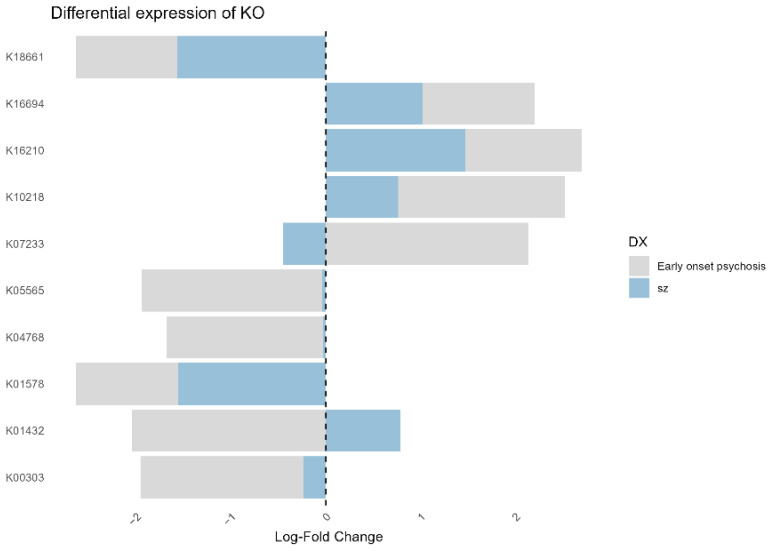
Barplot displaying the differential expression (log-fold change) of KEGG Orthology (KO) terms in patients with early-onset psychosis and schizophrenia compared to non-psychotic. The log-fold change reflects the relative abundance of KO terms between groups.

Regarding the gene orthology analysis (KO), a total of 10 differentially expressed genes were identified. A lower abundance of the genes K18661, K01578, K00303, K05565, and K04768 was observed in patients with schizophrenia and early-onset psychosis. On the other hand, the genes K16694, K16210, and K10218 showed higher abundance in both patient groups compared to the non-psychotic group. The gene K07233 was found to be increased in patients with early-onset psychosis and decreased in patients with schizophrenia compared to the non-psychotic group. Finally, the gene K01432 showed an increase in patients with schizophrenia and a decrease in patients with early-onset psychosis compared to individuals without psychosis (Figure 7). No significant differences were found in the relative abundance of the MetaCyc pathways.

## 4. Discussion

The gut microbiome through the gut–brain axis has demonstrated that gut bacteria and their metabolites can influence the brain and mental health. Several studies have demonstrated the association of the gut microbiota and depression, anorexia, and schizophrenia [27,28].

In our study, in addition to evaluating the presence/absence of bacteria in each group (observed index), we also assessed the Simpson, Shannon, and Chao1 indices. These indices were chosen for their respective strengths in measuring microbial diversity, particularly in communities characterized by a large number of species with relatively low abundances [29,30]. The Shannon index is particularly useful because it accounts for both species richness and their relative abundances, providing a comprehensive view of community structure [29].

We also employed the Chao1 index, which estimates total richness by considering both observed species and those that may be present at low abundance but are not sampled [30]. By incorporating these indices, we aimed to capture the complexities of microbial diversity and provide a more robust evaluation of community structure. In this study, we found that patients with schizophrenia showed a significantly higher alpha diversity compared to non-psychotic patients and those with early-onset psychosis, according to the Shannon index. It is possible that this higher diversity is at least partly due to the age differences between the groups, as the patients with schizophrenia were adults, whereas the other two groups were adolescents, with a mean difference in age of 22 years. There are a couple of possible explanations for this higher diversity. On the one hand, microbial distributions can be age-specific, or we can identify microbial signatures associated with aging, just as species diversity increases with age, which could correlate with what was observed in this study [31,32]. On the other hand, since patients with schizophrenia were taking antipsychotics at the time of stool collection, drug treatment affects bacterial abundance, as recently reported by Stiernborg et al., showing an increase in alpha diversity in patients with schizophrenia taking antipsychotics [33,34].

We did not observe differences in alpha diversity measures between patients with early-onset psychosis and non-psychotic controls. This may reflect the complexity of microbial community dynamics, where taxonomic differences do not always correlate with changes in diversity indices. This finding suggests that while certain taxa may vary in abundance, the overall richness and evenness of microbial communities in these groups may remain comparable, and this balance could be crucial in maintaining individual homeostasis.

Regarding beta diversity, we found statistically significant differences between the three study groups suggesting that while diagnosis may explain part of the gut bacteria variability, other factors such as age or the use of antipsychotics may also contribute to this variability.

When comparing the relative abundance of genera across the three groups, using MaAsLin2, we found that *Desulfovibrionaceae Incertae Sedis*, *Paraprevotella*, *UCG_010*, *Oscillospiraceae UCG-005*, *Oscillospiraceae UCG-003*, *Oscillospiraceae UCG-002*, *Oscillospiraceae NK4A214*, *Coprobacter*, and *Coprobacillus* were significantly more abundant in patients with schizophrenia compared to non-psychotic controls (Figure 5).

Desulfovibrionaceae is a family of anaerobic sulfate-reducing bacteria that have been associated with inflammatory bowel disease (IBD). These bacteria can disrupt the tight junctions of the intestinal barrier, leading to increased permeability [35]. In patients with schizophrenia, impaired intestinal barrier integrity is well-documented, allowing bacterial translocation to the lamina propria and triggering a pro-inflammatory cytokine cascade [36,37]. Similarly, *Paraprevotella* is a genus associated with depression, known for producing succinic acid, which stimulates interleukin-1β production. This interleukin-1β has been shown to enhance the synthesis of IDO1, facilitating the conversion of tryptophan to kynurenine—a pathway that has been altered in patients with schizophrenia [38,39,40,41]. Furthermore, pro-inflammatory cytokines may circulate through the bloodstream and potentially cross the blood–brain barrier, leading to microglial activation and triggering an inflammatory response in the central nervous system, thereby increasing the risk of developing schizophrenia [13,42].

In contrast, the family Oscillospiraceae, previously known as Ruminnococcaceae, has been linked to increased butyrate production and a reduction in depressive symptoms. Studies in patients with schizophrenia have shown that decreased levels of these bacteria correlate with heightened negative symptoms [43,44]. Notably, *Ruminnococcaceae_UCG_005* has been linked to claudin, a protein essential for maintaining the integrity of tight junctions in the intestinal barrier [45]. The elevated abundance of this family in our schizophrenia patients may be attributable to long-term medication use, suggesting that the increase in Oscillospiraceae could be a result of pharmacological treatment and effective disease management.

In addition to the families that influence intestinal permeability, we found that *Coprobacillus* and *Coprobacter* were elevated in patients with schizophrenia in our study. Both genera have been associated with cognitive impairment, with *Coprobacillus* specifically reported at higher levels in individuals with schizophrenia. Although it has been proposed that the effects of *Coprobacillus* may be mediated by appetite-regulating hormones, the exact mechanisms by which these bacteria contribute to cognitive impairment remain unclear [37,45,46].

In contrast, we observed a decrease in the genus *Dorea* in patients with schizophrenia. Dorea is known for its role in producing short-chain fatty acids (SCFAs) and its anti-inflammatory properties [32]. Since schizophrenia is often associated with a pro-inflammatory state, the reduction of these bacteria aligns with the inflammatory profile of the disease [47].

In the comparison between patients with early-onset psychosis and the non-psychotic group, the genus *Staphylococcus* was found to be significantly reduced in the early-onset psychosis group (MaAsLin2, *p* < 0.05) (Figure 5). While *Staphylococcus* is commonly associated with infection and inflammation, recent studies suggest its potential role as a serotonin producer in the gut [48,49]; however, its reduction in this study suggests a potential deviation from the expected inflammatory profile. Further studies with larger cohorts are needed to determine whether this decrease is specific to early-onset psychosis and to explore the underlying mechanisms, as there are currently no reports indicating alterations of this genus in early-onset psychosis or schizophrenia.

On the other hand, it is also essential to consider the potential impact of pharmacological treatments on microbial composition. In our study, we observed several genera associated with the use of atypical antipsychotics, valproate, and sertraline. For instance, *Prevotellaceae Leyella y Prevotellaceae Incertae Sedis* were significantly correlated with atypical antipsychotic use. *Leyella*, also known as *Prevotella*, has been linked to positive emotional states and is recognized for its production of short-chain fatty acids such as acetate, propionate, and butyrate [49,50]. This is particularly relevant given the therapeutic effects of these antipsychotics, which may correlate with the increased production of butyrate, suggesting a possible role in supporting gut health and alleviating both positive and negative symptoms in patients with schizophrenia [51].

Additionally, in patients taking sertraline, we noted an increase in the genus *Weissella.* A study conducted on mice administered *Weissella paramesenteroid* WpK4 found that it helped reduce symptoms of depression and anxiety, as well as lower levels of cytokines such as interleukin-1β and IL-6. By decreasing these interleukins involved in the synthesis of the enzyme IDO1, this could help regulate the tryptophan–kynurenine pathway. As a result, instead of being metabolized to kynurenine, tryptophan may be synthesized into serotonin, potentially improving symptoms in patients with schizophrenia [40].

Moreover, patients receiving valproate treatment exhibited a decrease in *Desulfovibrionaceae Incertae Sedis*, which aligns with its known association with intestinal permeability. This suggests that valproate may contribute to stabilizing the intestinal barrier. Furthermore, we observed an increase in *Kandleria* and *Howardella*. *Kandleria* has been linked to butyrate production, while *Howardella* has been found in lower abundance in patients with depression. Thus, the increase in these genera may be advantageous for alleviating the negative symptoms associated with schizophrenia [41,52].

The differential abundance of these bacteria contributes to the characterization of the gut microbiota in patients with psychosis. This characterization may potentially aid in the identification of beneficial bacteria to propose future dietary interventions. Nevertheless, it is important to highlight that there are several other factors, including diet, type of birth, the host genome, and the environment with a relevant impact on the disease and on the gut microbiota [53,54]. For example, studies comparing the non-western diet with the western diet have found that the richness of fatty acid-producing bacteria is higher in individuals with the former diet, because in part this diet could help prevent the establishment of some pathogenic bacteria [55].

The relationship between the diet and the composition of the gut microbiota is particularly relevant in Mexico, where the diet of children living in Mexico City is a western diet characterized by a high intake of animal protein, refined vegetable oils, grains, and sugars (soft drinks, snacks, cookies, etc.), and a low intake of vegetables and fiber [53]. The study design did not allow us to collect dietary information, thus limiting our ability to seek potential associations between the gut microbiome, early-onset psychosis, schizophrenia, and diet. Future studies considering dietary and environmental factors are warranted to gain a broader understanding of the underlying associations of diet, the gut microbiota, early-onset psychosis, and schizophrenia.

According to the PICRUSt2 analysis, we observed a significant decrease in the abundance of malonyl-CoA decarboxylase (EC 4.1.1.9) in patients with schizophrenia and early-onset psychosis, which was also reflected in the reduced expression of the K01578 gene. This enzyme plays a crucial role in fatty acid metabolism, and lower levels have been associated with cognitive impairment [56]. In patients with schizophrenia, it would be important to investigate whether a deficiency in this enzyme could be linked to cognitive deficits, suggesting a potential area for future research into the metabolic underpinnings of cognitive dysfunction in these patients.

Similarly, glycine oxidase (EC 1.4.3.19) catalyzes the oxidation of glycine, an amino acid that acts as an inhibitory neurotransmitter in the brain. It has been reported that glycine levels are elevated in patients with schizophrenia [57]. A reduction in glycine oxidase activity could contribute to this accumulation of glycine in the system, which may be associated with the exacerbation of symptoms in these patients. However, further studies are necessary to investigate the potential relationship between glycine oxidase activity and glycine levels in individuals with schizophrenia.

Additionally, the increased expression of gene K01432 could be an additional factor influencing the regulation of tryptophan metabolism in patients with schizophrenia, considering that, as previously mentioned, this metabolic pathway is dysregulated in these patients.

## 5. Conclusions

In summary, our analysis highlights the intricate relationship between the gut microbiota, metabolic pathways, and psychiatric conditions such as schizophrenia and early-onset psychosis. The differential abundance of specific bacteria contributes to the characterization of the gut microbiota in these patients, potentially guiding future prebiotics, probiotics, and dietary interventions. Notably, pharmacological treatments, including atypical antipsychotics, valproate, and sertraline, appear to influence microbial composition, further complicating this dynamic. Additionally, the dysregulation of metabolic pathways, particularly those involving tryptophan and fatty acid metabolism, underscores the need for further investigation into the metabolic underpinnings of cognitive dysfunction in schizophrenia. Future studies should aim to explore these associations between diet, gut microbiota, and mental health.

## Figures and Tables

**Table 1:** Clinical characteristics of non-psychosis subjects, patients with early-onset psychosis, and patients with schizophrenia. Values are presented as mean ± SD or ratio.

	Non-Psychosis (*n* = 21)	Early Onset Psychosis (*n* = 12)	Schizophrenia (*n* = 15)	*p* Value
Age ± sd	14.05 ± 2.83	14.84 ± 1.95	36.57 ± 9.22	<0.01
Sex ratio (F/M)	9/12	6/6	5/10	0.67
BMI (kg/m^2^ ± sd)	24.85 ± 4.84	24.27 ± 4.45	28.33 ± 6.80	0.13
Years of education (years ± sd)	8.0 ± 2.82	8.66 ± 2.3	11.28 ± 2.46	<0.01

## Data Availability

The data supporting the findings of this study are available in the Zenodo repository at https://zenodo.org/records/13931680, accessed on 14 October 2024. This dataset includes fecal microbiota sequencing data from adolescents with early-onset psychosis, adults with schizophrenia, and non-psychotic controls, analyzed using QIIME2 and PICRUSt2.

## Supplementary Materials

The following supporting information can be downloaded at: https://www.mdpi.com/article/10.3390/microorganisms12102071/s1. Figure S1. Alpha diversity of the microbiota in patients with schizophrenia compared to non-psychotic individuals and early-onset psychosis patients. The indices of Observed, Chao1, and Simpson diversity are presented. Table S1. Result of Wilcoxon test between groups from Shannon alpha diversity. Table S2. Pairwise PERMANOVA results comparing Bray–Curtis distances between groups. “**” indicates significance at an alpha of 0.01. Df, degrees of freedom; SumsOfSqs sum of squares; F.model, F value by permutation, p.values based on 999 permutations; p.adj, p adjust; sig, significance. Table S3. Pairwise PERMANOVA results comparing Jaccard distances between groups. “**” indicates significance at an alpha of 0.01. Df, degrees of freedom; SumsOfSqs sum of squares; F.model, F value by permutation, p.values based on 999 permutations; p.adj, p adjust; sig, significance. Table S4. Pairwise PERMANOVA results comparing Weighted Frac distances between groups. “**” indicates significance at an alpha of 0.01. Df, degrees of freedom; SumsOfSqs sum of squares; F.model, F value by permutation, p.values based on 999 permutations; p.adj, p adjust; sig, significance. Table S5. Pairwise PERMANOVA results comparing Unweighted Frac distances between groups. “**” indicates significance at an alpha of 0.01. Df, degrees of freedom; SumsOfSqs sum of squares; F.model, F value by permutation, p.values based on 999 permutations; p.adj, p adjust; sig, significance.

## References

[1] American Psychiatric Association, American Psychiatric Association (2013). Diagnostic and Statistical Manual of Mental Disorders: DSM-5.

[2] (1992). World Health Organization The ICD-10 Classification of Mental and Behavioural Disorders: Clinical Descriptions and Diagnostic Guidelines.

[3] McClellan J. (2018). Psychosis in Children and Adolescents. J. Am. Acad. Child Adolesc. Psychiatry.

[4] Long J., Huang G., Liang W., Liang B., Chen Q., Xie J., Jiang J., Su L. (2014). The Prevalence of Schizophrenia in Mainland China: Evidence from Epidemiological Surveys. Acta Psychiatr. Scand..

[5] Zwicker A., Denovan-Wright E.M., Uher R. (2018). Gene–Environment Interplay in the Etiology of Psychosis. Psychol. Med..

[6] Delphin N., Aust C., Griffiths L., Fernandez F. (2024). Epigenetic Regulation in Schizophrenia: Focus on Methylation and Histone Modifications in Human Studies. Genes.

[7] Singh J., Vanlallawmzuali, Singh A., Biswal S., Zomuansangi R., Lalbiaktluangi C., Singh B.P., Singh P.K., Vellingiri B., Iyer M. (2024). Microbiota-Brain Axis: Exploring the Role of Gut Microbiota in Psychiatric Disorders–A Comprehensive Review. Asian J. Psychiatry.

[8] Damiani F., Cornuti S., Tognini P. (2023). The Gut-Brain Connection: Exploring the Influence of the Gut Microbiota on Neuroplasticity and Neurodevelopmental Disorders. Neuropharmacology.

[9] Costea P.I., Hildebrand F., Arumugam M., Bäckhed F., Blaser M.J., Bushman F.D., de Vos W.M., Ehrlich S.D., Fraser C.M., Hattori M. (2018). Enterotypes in the Landscape of Gut Microbial Community Composition. Nat. Microbiol..

[10] Zimmermann M., Zimmermann-Kogadeeva M., Wegmann R., Goodman A.L. (2019). Separating Host and Microbiome Contributions to Drug Pharmacokinetics and Toxicity. Science.

[11] Adak A., Khan M.R. (2019). An Insight into Gut Microbiota and Its Functionalities. Cell Mol. Life Sci..

[12] Nuncio-Mora L., Lanzagorta N., Nicolini H., Sarmiento E., Ortiz G., Sosa F., Genis-Mendoza A.D. (2023). The Role of the Microbiome in First Episode of Psychosis. Biomedicines.

[13] Li Z., Tao X., Wang D., Pu J., Liu Y., Gui S., Zhong X., Yang D., Zhou H., Tao W. (2024). Alterations of the Gut Microbiota in Patients with Schizophrenia. Front Psychiatry.

[14] Chan M.K., Cooper J.D., Bahn S. (2015). Commercialisation of Biomarker Tests for Mental Illnesses: Advances and Obstacles. Trends Biotechnol..

[15] Bolyen E., Rideout J.R., Dillon M.R., Bokulich N.A., Abnet C.C., Al-Ghalith G.A., Alexander H., Alm E.J., Arumugam M., Asnicar F. (2019). Reproducible, Interactive, Scalable and Extensible Microbiome Data Science Using QIIME 2. Nat. Biotechnol..

[16] Callahan B.J., McMurdie P.J., Rosen M.J., Han A.W., Johnson A.J.A., Holmes S.P. (2016). DADA2: High-Resolution Sample Inference from Illumina Amplicon Data. Nat. Methods.

[17] Katoh K., Misawa K., Kuma K., Miyata T. (2002). MAFFT: A Novel Method for Rapid Multiple Sequence Alignment Based on Fast Fourier Transform. Nucleic Acids Res..

[18] McMurdie P.J., Holmes S. (2013). Phyloseq: An R package for reproducible interactive analysis and graphics of microbiome census data. PLoS ONE.

[19] Oksanen J., Simpson G.L., Blanchet F.G., Kindt R., Legendre P., Minchin P.R., O’Hara R.B., Solymos P., Stevens M.H.H., Szoecs E. Vegan: Community Ecology Package 2024. https://CRAN.R-project.org/package=vegan.

[20] R: The R Project for Statistical Computing. https://www.r-project.org/.

[21] Robeson M., O’Rourke D., Kaehler B., Ziemski M., Dillon M., Foster J., Bokulich N. (2020). RESCRIPt: Reproducible Sequence Taxonomy Reference Database Management for the Masses. bioRxiv.

[22] Bokulich N.A., Kaehler B.D., Rideout J.R., Dillon M., Bolyen E., Knight R., Huttley G.A., Gregory Caporaso J. (2018). Optimizing Taxonomic Classification of Marker-Gene Amplicon Sequences with QIIME 2’s Q2-Feature-Classifier Plugin. Microbiome.

[23] Mallick H., Rahnavard A., McIver L.J., Ma S., Zhang Y., Nguyen L.H., Tickle T.L., Weingart G., Ren B., Schwager E.H. (2021). Multivariable Association Discovery in Population-Scale Meta-Omics Studies. PLOS Comput. Biol..

[24] Langille M.G.I., Zaneveld J., Caporaso J.G., McDonald D., Knights D., Reyes J.A., Clemente J.C., Burkepile D.E., Vega Thurber R.L., Knight R. (2013). Predictive Functional Profiling of Microbial Communities Using 16S rRNA Marker Gene Sequences. Nat. Biotechnol..

[25] Analysis of Compositions of Microbiomes with Bias Correction | Nature Communications. https://www.nature.com/articles/s41467-020-17041-7.

[26] Linear and Nonlinear Correlation Estimators Unveil Undescribed Taxa Interactions in Microbiome Data | Nature Communications. https://www.nature.com/articles/s41467-022-32243-x.

[27] Vasileva S.S., Yang Y., Baker A., Siskind D., Gratten J., Eyles D. (2024). Associations of the Gut Microbiome with Treatment Resistance in Schizophrenia. JAMA Psychiatry.

[28] Akhondzadeh S. (2019). Microbiome and Schizophrenia. Avicenna J. Med. Biotechnol..

[29] Chao A., Shen T.-J. (2003). Nonparametric Estimation of Shannon’s Index of Diversity When There Are Unseen Species in Sample. Environ. Ecol. Stat..

[30] Chao A. (1984). Nonparametric Estimation of the Number of Classes in a Population. Scand. J. Stat..

[31] Kim J.-W., Lee J.S., Kim J.H., Jeong J.-W., Lee D.H., Nam S. (2018). Comparison of Microbiota Variation in Korean Healthy Adolescents with Adults Suggests Notable Maturity Differences. OMICS J. Integr. Biol..

[32] Wang H., Chen Y., Feng L., Lu S., Zhu J., Zhao J., Zhang H., Chen W., Lu W. (2024). A Gut Aging Clock Using Microbiome Multi-View Profiles Is Associated with Health and Frail Risk. Gut Microbes.

[33] Stiernborg M., Prast-Nielsen S., Melas P.A., Skott M., Millischer V., Boulund F., Forsell Y., Lavebratt C. (2024). Differences in the Gut Microbiome of Young Adults with Schizophrenia Spectrum Disorder: Using Machine Learning to Distinguish Cases from Controls. Brain Behav. Immun..

[34] Xu Y., Shao M., Fang X., Tang W., Zhou C., Hu X., Zhang X., Su K.-P. (2022). Antipsychotic-Induced Gastrointestinal Hypomotility and the Alteration in Gut Microbiota in Patients with Schizophrenia. Brain Behav. Immun..

[35] Singh S.B., Carroll-Portillo A., Lin H.C. (2023). Desulfovibrio in the Gut: The Enemy Within?. Microorganisms.

[36] Sung K., Zhang B., Wang H.E., Bai Y., Tsai S., Su T., Chen T., Hou M., Lu C., Wang Y. (2022). Schizophrenia and Risk of New-onset Inflammatory Bowel Disease: A Nationwide Longitudinal Study: Alimentary Pharmacology & Therapeutics. Aliment. Pharmacol. Ther..

[37] Misiak B., Pawlak E., Rembacz K., Kotas M., Żebrowska-Różańska P., Kujawa D., Łaczmański Ł., Piotrowski P., Bielawski T., Samochowiec J. (2024). Associations of Gut Microbiota Alterations with Clinical, Metabolic, and Immune-Inflammatory Characteristics of Chronic Schizophrenia. J. Psychiatr. Res..

[38] Zheng P., Zeng B., Liu M., Chen J., Pan J., Han Y., Liu Y., Cheng K., Zhou C., Wang H. (2019). The Gut Microbiome from Patients with Schizophrenia Modulates the Glutamate-Glutamine-GABA Cycle and Schizophrenia-Relevant Behaviors in Mice. Sci. Adv..

[39] Wang Z., Yuan X., Zhu Z., Pang L., Ding S., Li X., Kang Y., Hei G., Zhang L., Zhang X. (2023). Multiomics Analyses Reveal Microbiome–Gut–Brain Crosstalk Centered on Aberrant Gamma-Aminobutyric Acid and Tryptophan Metabolism in Drug-Naïve Patients with First-Episode Schizophrenia. Schizophr. Bull..

[40] Sandes S., Figueiredo N., Pedroso S., Sant’Anna F., Acurcio L., Abatemarco Junior M., Barros P., Oliveira F., Cardoso V., Generoso S. (2020). *Weissella Paramesenteroides* WpK4 Plays an Immunobiotic Role in Gut-Brain Axis, Reducing Gut Permeability, Anxiety-like and Depressive-like Behaviors in Murine Models of Colitis and Chronic Stress. Food Res. Int..

[41] Barandouzi Z.A., Starkweather A.R., Henderson W.A., Gyamfi A., Cong X.S. (2020). Altered Composition of Gut Microbiota in Depression: A Systematic Review. Front. Psychiatry.

[42] Gutiérrez-Calabrés E., Ortega-Hernández A., Modrego J., Gómez-Gordo R., Caro-Vadillo A., Rodríguez-Bobada C., González P., Gómez-Garre D. (2020). Gut Microbiota Profile Identifies Transition From Compensated Cardiac Hypertrophy to Heart Failure in Hypertensive Rats. Hypertension.

[43] Liang S., Sin Z.Y., Yu J., Zhao S., Xi Z., Bruzzone R., Tun H.M. (2022). Multi-Cohort Analysis of Depression-Associated Gut Bacteria Sheds Insight on Bacterial Biomarkers across Populations. Cell Mol. Life Sci..

[44] Nguyen T.T., Kosciolek T., Maldonado Y., Daly R.E., Martin A.S., McDonald D., Knight R., Jeste D.V. (2019). Differences in Gut Microbiome Composition between Persons with Chronic Schizophrenia and Healthy Comparison Subjects. Schizophr. Res..

[45] Duan M., Liu F., Fu H., Lu S., Wang T. (2021). Preoperative Microbiomes and Intestinal Barrier Function Can Differentiate Prodromal Alzheimer’s Disease From Normal Neurocognition in Elderly Patients Scheduled to Undergo Orthopedic Surgery. Front. Cell Infect. Microbiol..

[46] Zhou K., Baranova A., Cao H., Sun J., Zhang F. (2024). Gut Microbiome and Schizophrenia: Insights from Two-Sample Mendelian Randomization. Schizophr.

[47] Ahmed G.K., Ramadan H.K.-A., Elbeh K., Haridy N.A. (2024). The Role of Infections and Inflammation in Schizophrenia: Review of the Evidence. Middle East Curr. Psychiatry.

[48] Liu A., Garrett S., Hong W., Zhang J. (2024). Staphylococcus Aureus Infections and Human Intestinal Microbiota. Pathogens.

[49] Miri S., Yeo J., Abubaker S., Hammami R. (2023). Neuromicrobiology, an Emerging Neurometabolic Facet of the Gut Microbiome?. Front. Microbiol..

[50] Zhou X., Ganz A.B., Rayner A., Cheng T.Y., Oba H., Rolnik B., Lancaster S., Lu X., Li Y., Johnson J.S. (2024). Dynamic Human Gut Microbiome and Immune Shifts During an Immersive Psychosocial Therapeutic Program. bioRxiv.

[51] Li X., Yuan X., Pang L., Zhang S., Li Y., Huang X., Fan X., Song X. (2022). The Effect of Serum Lipids and Short-Chain Fatty Acids on Cognitive Functioning in Drug-Naïve, First Episode Schizophrenia Patients. Psychiatry Res..

[52] Wang J., Zhang Y., Wang X., Li F., Zhang D., Li X., Zhao Y., Zhao L., Xu D., Cheng J. (2022). Association between Rumen Microbiota and Marbling Grade in Hu Sheep. Front. Microbiol..

[53] Sánchez-Quinto A., Cerqueda-García D., Falcón L.I., Gaona O., Martínez-Correa S., Nieto J., G-Santoyo I. (2020). Gut Microbiome in Children from Indigenous and Urban Communities in México: Different Subsistence Models, Different Microbiomes. Microorganisms.

[54] Hollister E.B., Riehle K., Luna R.A., Weidler E.M., Rubio-Gonzales M., Mistretta T.-A., Raza S., Doddapaneni H.V., Metcalf G.A., Muzny D.M. (2015). Structure and Function of the Healthy Pre-Adolescent Pediatric Gut Microbiome. Microbiome.

[55] De Filippo C., Cavalieri D., Di Paola M., Ramazzotti M., Poullet J.B., Massart S., Collini S., Pieraccini G., Lionetti P. (2010). Impact of Diet in Shaping Gut Microbiota Revealed by a Comparative Study in Children from Europe and Rural Africa. Proc. Natl. Acad. Sci. USA.

[56] Zhang J.M., Hao L.L., Qiu W.J., Zhang H.W., Chen T., Ji W.J., Zhang Y., Liu F., Gu X.F., Yang S.H. (2024). Clinical, Biochemical and Genetic Characteristics and Long-Term Follow-up of Five Patients with Malonyl-CoA Decarboxylase Deficiency. Brain Dev..

[57] Pei J.-C., Luo D.-Z., Gau S.-S., Chang C.-Y., Lai W.-S. (2021). Directly and Indirectly Targeting the Glycine Modulatory Site to Modulate NMDA Receptor Function to Address Unmet Medical Needs of Patients With Schizophrenia. Front. Psychiatry.

