# Supplementary material for: Comparative Analysis of Fecal Microbiota Between Adolescents with Early-Onset Psychosis and Adults with Schizophrenia
Source: Microorganisms. 2024 Oct 16;12(10):2071. doi: 10.3390/microorganisms12102071 (PMC11510430; doi:10.3390/microorganisms12102071)
Supplement: Supplementary file 1 [file microorganisms-12-02071-s001.zip › microorganisms-3218385-supplementary.pdf]

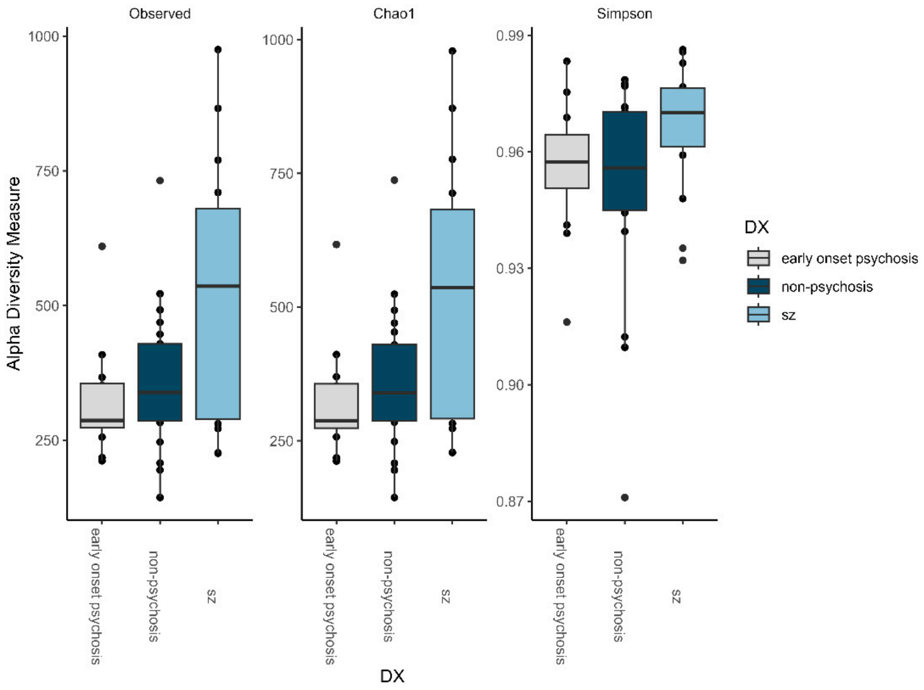

Figure S1. Alpha diversity of the microbiota in patients with schizophrenia compared to non-psychotic individuals and early-onset psychosis patients. The indices of Observed, Chao1, and Simpson diversity are presented.

Table S1. Result of Wilcoxon test between groups from Shannon alpha diversity

**Group 1**	**Group 2**	**p.adj**
Non-psychosis	Early onset psychosis	0.4486
SZ	Early onset psychosis	0.0321
SZ	Non-psychosis	0.0029

Table S2. Pairwise PERMANOVA results comparing Bray–Curtis distances between groups. "**" indicates significance at an alpha of 0.01. Df, degrees of freedom; SumsOfSqs sum of squares; F.model, F value by permutation, p.values based on 999 permutations; p.adj, p adjust; sig, significance.

	**Df**	**SumsOfSqs**	**F.Model**	**R2**	**p.value**	**p.adj**	**sig**
SZ vs Non-psychosis	1	0.6306994	2.17444039	0.06010986	4.00E-04	0.0012	**
SZ vs Early onset psychosis	1	0.37134496	1.30954892	0.04977466	0.0851	0.0922	
		0.3460879					

	Df	SumsOfSqs	F.Model	R2	p.value	p.adj	sig
Non-psychosis vs Early onset psychosis	1		1.26354876	0.03916335	0.0922	0.0922	

Table S3. Pairwise PERMANOVA results comparing Jaccard distances between groups. "**" indicates significance at an alpha of 0.01. Df, degrees of freedom; SumsOfSqs sum of squares; F.model, F value by permutation, p.values based on 999 permutations; p.adj, p adjust; sig, significance.

	**Df**	**SumsOfSqs**	**F.Model**	**R2**	**p.value**	**p.adj**	**sig**
SZ vs Non-psychosis	1	0.59482689	1.39373099	0.03937791	3.00E-04	9.00E-04	**
SZ vs Early onset psychosis	1	0.4783083	1.1274592	0.04315227	0.0624	0.0936	
Non-psychosis vs Early onset psychosis	1	0.44691626	1.06451806	0.03319925	0.1508	0.1508	

Table S4. Pairwise PERMANOVA results comparing Weighted Frac distances between groups. "**" indicates significance at an alpha of 0.01. Df, degrees of freedom; SumsOfSqs sum of squares; F.model, F value by permutation, p.values based on 999 permutations; p.adj, p adjust; sig, significance

	**Df**	**SumsOfSqs**	**F.Model**	**R2**	**p.value**	**p.adj**	**sig**
SZ vs Non-psychosis	1	0.00060883	2.10995079	0.05843128	0.0015	0.0045	**
SZ vs Early onset psychosis	1	0.00071177	2.00840407	0.07436219	0.0045	0.00675	**
Non-psychosis vs Early onset psychosis	1	0.00047659	1.46591053	0.0451523	0.1053	0.1053	

Table S5. Pairwise PERMANOVA results comparing Unweighted Frac distances between groups. “**” indicates significance at an alpha of 0.01. Df, degrees of freedom; SumsOfSqs sum of squares; F.model, F value by permutation, p.values based on 999 permutations; p.adj, p adjust; sig, significance

	**Df**	**SumsOfSqs**	**F.Model**	**R2**	**p.value**	**p.adj**	**sig**
SZ vs Non-psychosis	1	0.5200997	1.20534908	0.03423767	0.0018	0.00405	**
SZ vs Early onset psychosis	1	0.51666314	1.21683914	0.04641441	0.0027	0.00405	**
Non-psychosis vs Early onset psychosis	1	0.44679431	1.05349682	0.03286683	0.0886	0.0886	
